# Perspectives on Pyrochlores, Defect Fluorites, and Related Compounds: Building Blocks for Chemical Diversity and Functionality

**DOI:** 10.3389/fchem.2021.778140

**Published:** 2021-11-12

**Authors:** Gregory R. Lumpkin, Robert D. Aughterson

**Affiliations:** Australian Nuclear Science and Technology Organisation, Sydney, NSW, Australia

**Keywords:** defect fluorite, modulated structures, pyrochlore, weberite, zirconolite

## Abstract

In this article we provide some perspectives on a range of pyrochlore and defect fluorite type compounds with nominal A_2_B_2_O_7_, A_2_BO_5_, ABC_2_O_7_, and other stoichiometries. Typically, the phase transformations and stability fields in these systems are mapped as a function of the ionic radii of the A and B-site cations, e.g., the A/B cation radius ratio (r_A_/r_B_). This provides a useful guide to compatible structures and compositions for the development of advanced materials. Pyrochlore commonly transforms to a defect fluorite structure at high temperature in many systems; however, it is not uncommon to observe defect fluorite as the initial metastable phase at low temperature. The patterns of order-disorder observed in these materials are primarily due to the energetics of layer stacking, the defect formation and migration energies of cations and anions, or modulations of the parent cubic structure in 3 + *n* dimensional space. The first lead to predominantly non-cubic derivatives of the parent defect fluorite structure (e.g., zirconolite polytypes), the second control the order-disorder processes, and the latter lead to a variety of subtle additional scattering features within the cubic parent structure. Although the energetics of cation disorder and anion-vacancy disorder have become more accessible via atomistic approaches (e.g., MD and DFT), we continue to find interesting physical-chemical problems in these materials. For example, although there are significant differences in composition (Tb/Zr ratio and O content) between Tb_2_Zr_2_O_7_ and Tb_2_ZrO_5_, both of which are defect fluorites, we note that the modulations found in these two compounds by electron scattering are virtually identical with regard to the direction and magnitude of displacement from the normal Bragg diffracted beams. This suggests that neither the A/B cation ratio nor the oxygen stoichiometry have a significant effect on the modulations. The general observations on the systems of compounds noted in this paper rest primarily in the context of industrial materials for nuclear waste disposal, potential applications in inert matrix fuel designs, and other important technological applications such as ionic conductivity, electrical conductivity, and magnetism. Scientific advances in these areas have been underpinned by recent advances in ion irradiation, synchrotron X-ray, neutron scattering, and modelling and simulation capabilities. Furthermore, there has been some renewed interest in natural samples, e.g., Th-U zirconolite and pyrochlore as analogues for potential host phases in nuclear waste forms. In particular, the natural pyrochlores have provided additional details with regard to radiation damage ingrowth, percolation transitions, and the relationships between accumulated dose and physical properties including hardness, elastic modulus. Specific details of the thermal annealing of these samples have also been elucidated in considerable detail.

## Introduction

Compounds based on the structure of fluorite, including pyrochlore, zirconolite, and related structure types with different stoichiometries (Table 1) are of interest for numerous technological applications, including nuclear fuel and related materials (e.g., inert matrix fuel), nuclear waste forms, fast ion conductors, and magnetic materials, among others. Starting with the basic fluorite structure, ^VIII^M^IV^X_2_, where M can be a range of medium to large tetravalent cations (e.g., Zr, Hf, Th, U) or divalent cations (e.g., Ca, Sr, Ba) in eightfold coordination and X is typically O^2−^ or F^−^ in fourfold coordination, there are many variations on the theme when considering charge balancing combinations of cations and anions. A major outcome of the crystal chemical flexibility of the fluorite structure type is the ability to incorporate monovalent and trivalent cations, in particular Y and rare earth elements, presenting a wealth of compositions and potentially useful properties. These structures exist in cubic space group *Fm3m* and when there is more than one cation present, they are generally disordered over the available cation sites; however, in many compositions electron diffraction patterns reveal the structures also include one or more modulations of the parent fluorite structure (Withers et al., 1991; Tabira et al., 2001). These modulated structures indicate the presence of chemical and/or displacive disorder within the underlying crystal structure. Furthermore, oxygen vacancies may be present if additional cations having lower valence states (e.g., Y^3+^ or Ca^2+^) are not charge balanced by higher valence cations (e.g., W^6+^ or Nb^5+^) with the cations, anions, and vacancies being disordered over the available lattice sites. These compounds are commonly referred to as defect fluorites.

**TABLE 1:** Some general aspects of ordered structure types and their related disordered, defect fluorite structures. Table is arranged by increasing M/X ratio from top to bottom.

Nomenclature	Space Group	Ordered	Disordered	M/X	X vacancies
Ideal Fluorite	*Fm*3*m*	n.a.	MX_2_	0.5000	0.0000
“227” Type Defect fluorite	*Fm*3*m*	n.a.	MX_1.75_	0.5714	0.2500
Pyrochlore	*Fd*3*m*	A_2_B_2_X_6_Y or A_3_BX_7_	MX_1.75_	0.5714	0.2500
*Layered perovskite* ^a^	*P*2 *or Cmc*2	A_2_B_2_X_7_	n.a.	0.5714	n.a.
Zirconolite 2M^b^	*C*2*/c* (+)	ABC_2_X_7_	MX_1.75_	0.5714	0.2500
Weberite 2O^c^	*Imma* (+)	A_2_B_2_X_7_ or A_3_BX_7_	MX_1.75_	0.5714	0.2500
Delta Phase	*R* 3	A_4_B_3_X_12_	MX_1.714_	0.5833	0.2857
“215” Type	*Fm*3*m*	A_2_BX_5_	MX_1.667_	0.6000	0.3333

a These compounds are not defect fluorites, but they do consist of perovskite layers alternating with A_2_O_3_ layers which have some topological resemblance to the fluorite structure. They are included for reference due to possible phase transitions to pyrochlore, etc.

b Plus numerous polytypes and space groups due to layer stacking variations, including 3T, 3O. 4M, and 6T. In general, layered structures may be complicated by the intergrowth of polytypes and/or stacking disorder. Incommensurate, modulated structures may be observed in the defect fluorite compounds, including 227 type, 317 type, delta phase, and 215 type compounds as a function of composition and overall stoichiometry. The X anion vacancies are given relative to the ideal fluorite structure.

c Plus numerous polytypes and space groups due to layer stacking variations, including 2O, 2M, 3T, 4M, 5M, 6M, 6T, 7M, and 8O.

Ordered derivatives of the defect fluorite structure include compounds with the A_2_B_2_O_7_ pyrochlore structure (“227” type, e.g., Subramanian et al., 1983; Chakoumakos 1984), the weberite group of layered compounds, consisting of “227” and “317” type compositions depending on valence states of the cations (Cai and Nino, 2009; Euchner et al., 2019), the A_3_BO_7_ defect fluorite structures (“317” type), and the A_2_BO_5_ (“215” type) compounds described by Lau et al. (2007); Shepelev and Petrova (2008); Lau et al. (2008); Aughterson et al., 2014; Aughterson et al., 2015; Aughterson et al., 2018b. Furthermore, the zirconolite structure types with the general formula ABC_2_O_7_ encompass a smaller group of layered compounds with non-cubic symmetry but based on a defect fluorite subcell. These compounds have useful properties including the ability to incorporate actinides, extreme chemical flexibility across three types of crystallographic sites, and very high resistance to dissolution. Together with pyrochlore, a range of zirconolite compositions have been developed and extensively tested as a major component of nuclear waste forms (e.g., Vance et al., 2002; Strachan et al., 2005, Strachan et al., 2008; Icenhower, et al., 2006). In these broad families there are numerous compounds that are known and possibly others yet to be synthesized in the laboratory, providing a fertile ground for continued discovery research in phase transitions and useful industrial properties. The high level of interest in these materials has been evidenced by numerous papers in the scientific literature. Many of these compounds already have important technological applications in areas including nuclear fuel, nuclear waste forms, fast ion conductors, magnetism, and other areas of materials science (Subramanian et al., 1983; van Dijk et al., 1983; Kutty et al., 1994; Wang et al., 1999; Risovany et al., 2000; Vassen et al., 2000; Wu et al., 2002; Lutique et al., 2003a; Lutique et al., 2003b; Shlyahktina et al., 2005; Bansal and Zhu, 2007; Shimamura et al., 2007; Radhakrishnan et al., 2011; Sattonnay et al., 2013; Maram et al., 2018; Euchner et al., 2019; Lyashenko et al., 2010).

## Structure Types

Several groups of ordered fluorite-related compounds are compared in Table 1 together with their alternative disordered formulations. Th**e** defect fluorite structure type is based upon the classical fluorite structure (*Fm3m*) with M^VIII^X^IV^_2_ stoichiometry wherein the Roman number superscripts refer to the coordination numbers of the cations and anions, respectively. In general, M is typically Ca, Sr, Ba, and Pb for the compounds with X = F or Zr, Hf, Th, U, Pu, and Cm when X = O. However, in order to accommodate cations of lower or higher than the average 4 + valence state, the actual compositions may be oxygen deficient or in excess and the general formula can be written as MX_2 ± x_. For oxides, this formula is consistent with charge compensation by fewer oxygen anions if the average cation valence state is less than 4.0, leading to anion vacancies in the structure and a reduction of the cation-anion coordination number. This is the typical situation for the compounds often referred to as defect fluorites. However, if the average valence state is greater than 4.0, then excess oxygen may be present, up to some stability limit. Most nuclear fuels are based on UO_2 + x_ wherein U^4+^ is the nominal starting valence state, but higher valence states (U^5+^ and/or U^6+^) may occur in spent fuel under oxidizing conditions, leading to the condition where x > 2. In Table 1 we list some of the structure types of interest by increasing M/X ratio from ideal fluorite (M/X = 0.5) to the “215” type compounds (M/X = 0.6). This also relates to an increasing number of X anion vacancies in the defect fluorite asymmetric unit, which may be relevant to some of the properties (e.g., ionic transport, radiation tolerance, etc.) of the different compounds.

Ordered oxide pyrochlores are described by the general formula A_2_B_2_X_6_Y (after Chakoumakos, 1984) which can be expanded to reveal the coordination environments and vacant sites in space group *Fd3m* relative to the underlying disordered MX_2_ fluorite basis in space group *Fm3m*. The ordered oxide pyrochlore form can be expressed as: ^VIII^A_2_^VI^B_2_^IV^X_6_^IV^Y, where the Roman numerals represent the coordination numbers of the A and B cations, and the X and Y anions. In pyrochlore, the A and B cations are both on fixed positions located at 16*d* and 16*c*, with the X anions located on 48*f* and the Y anions located on 8*b*. We note that natural pyrochlores are common in evolved igneous systems, e.g., carbonatites and granitic pegmatites. The minerals are typically described by the end members NaCaNb_2_O_6_F (pyrochlore), NaCaTa_2_O_6_F (microlite), CaUTi_2_O_7_ (betafite), and other “end-member” components such as Ln_2_Ti_2_O_7_ (Ln = lanthanides). Pyrochlore may be considered the quintessential “227-type” oxide compound and there are literally hundreds of compounds, both natural and synthetic, that exist in this structural configuration. Subramanian et al. (1983) provided one of the earliest comprehensive reviews of pyrochlore with extended discussion of potentially useful properties, including magnetism, electrical properties, and oxygen ion migration.

The structure of zirconolite can be thought of as a pyrochlore that is compressed normal to one set of (111) planes, resulting in a layered structure in the prototype space group *C*2/*c* which refers to a 2-layered structure. Nevertheless, this structure retains evidence of the cubic defect fluorite subcell and may transform from monoclinic to cubic symmetry under irradiation during a crystalline to amorphous phase transformation. The composition of zirconolite is generally described by the general formula ABC_2_O_7_ wherein A = Ca, larger actinide and lanthanide cations, B = Zr, Ti, and smaller actinide and lanthanide cations, and C = Ti, Zr, Hf, Nb, Ta, Al, Mg, and transition metals. Various polytypes of zirconolite will form due to layer stacking sequences as a function of composition, including structures based on 3, 4, and 6-layer structural configurations.

The prototype for the weberite structure is orthorhombic, in space group *Imma*, has the 227-type stoichiometry, and the name was originally given to a mineral having the composition Na_2_MgAlF_7_ (Knop et al., 1982). As indicated in Table 1, the weberite structure is also adopted by some compounds with 317-type stoichiometry. For the compounds Y_3_TaO_7_, Gussev et al. (2020) noted that the space group of the long-range structure is consistent with either of two space groups, Ccmm or C222_1_, but using neutron pair distribution function (pdf) analysis, they demonstrated that the short-range structure in best represented in the latter space group. The crystal structure is derived from fluorite and is similar to the pyrochlore structure. The two structures have a similar cation sublattice with A cations in 8-fold coordination and B cations in six-fold coordination and built around (111) layers having the hexagonal tungsten bronze topology. As summarized by Cai and Nino (2009), there exists broad compositional flexibility in the weberite structure in both fluoride and oxide compounds. In oxide weberites, the A-sites are typically occupied by Na, K, Ag, Ca, Mn, Cd, Sr, Ba, Y, and lanthanides, whereas the B-sites are home to V, Sb, Te, Nb, Ta, Os, Bi, and U. In the latter two cases, the weberite compounds are Sr_2_Bi_2_O_7_ and Ba_2_U_2_O_7_ in which the B-site cations must be hexavalent by charge balance (Bi^6+^ and U^6+^) unless there are oxygen vacancies in the structure.

The delta phase refers to another group of lanthanide metal oxide compounds with rhombohedral symmetry (space group *R*3) and related to defect fluorite structures when disordered. These compounds are based on the general formula A_4_B_3_O_12_ wherein A = trivalent cations (e.g., Sc, Y and lanthanides) and B = tetravalent cations, primarily synthesized with B = Zr at this point in time. The composition of the delta phase can be reformulated to a pyrochlore type formula as: ^VIII^A_2_^VI^(A_0.286_B_1.714_)^IV^O_6_^IV^O_0.857_, and this equates to MX_1.714_ if represented as a disordered, defect fluorite structure. Compounds with the delta phase structure and stoichiometry are commonly encountered as intermediate compositions in some of the A-B metal oxide systems that have been studied to date.

Considerable recent interest in the A_2_BO_5_ or “215” structure types has been generated from studies of the phase relations for systems with A = Y and lanthanides and B = Ti (e.g., Shepelev and Petrova, 2006, 2008). When the A-site cation is Ho or other cations of similar radius, notably Y, or other cations from Dy to Er, a pyrochlore-like cubic structure forms with the nominal space group *Fd3m* (Lau et al., 2007, Lau et al., 2008; Aughterson et al., 2014). Although similar to the ordered structure of the A_2_B_2_X_6_Y pyrochlores, the pyrochlore-like A_2_BO_5_ structure is more complicated and as demonstrated using electron microscopy, the structure shows a tripling of along [111] directions and a less obvious, sevenfold repeat along the [622] directions when viewed down (110) in electron diffraction patterns. This incorporation of smaller lanthanide cations on the A-site leads to a transformation from the tripled pyrochlore structure to a disordered defect fluorite structure in these compounds.

**FIGURE 1:**
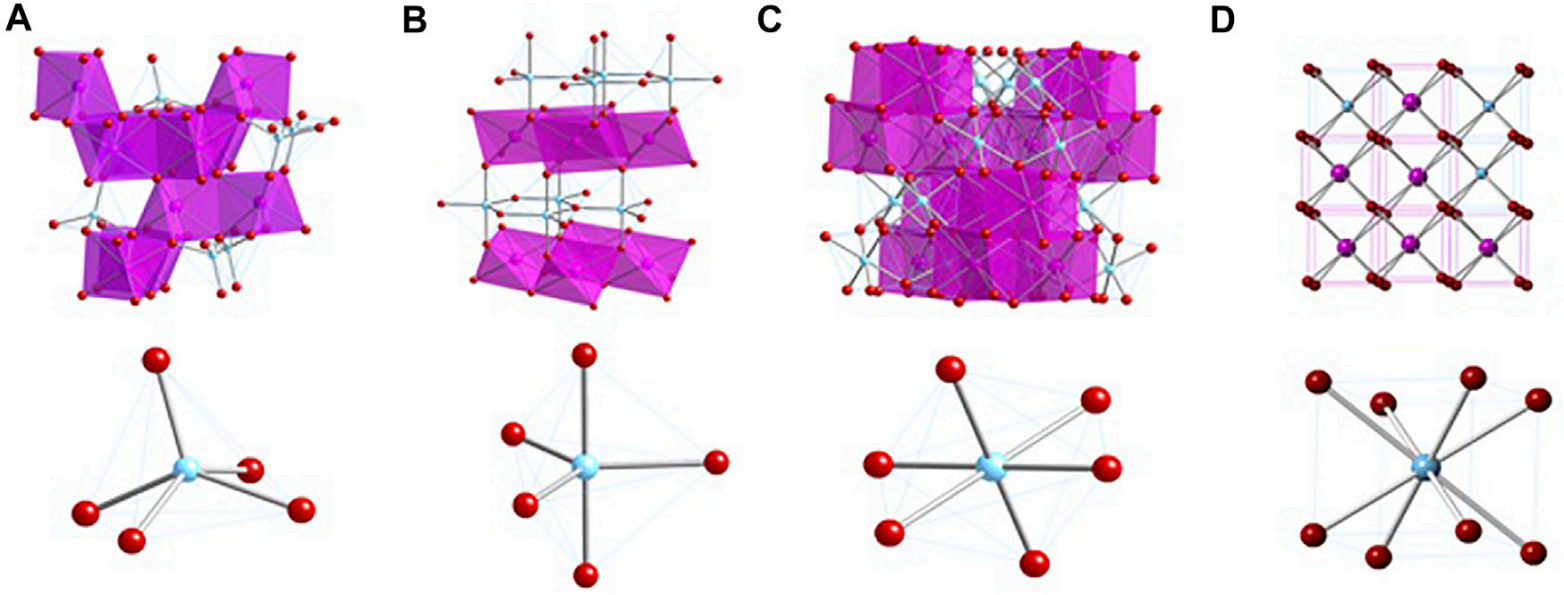
Crystal structures of the A_2_BO_5_ compounds with B = Ti. **(A)** Orthorhombic structure. **(B)** Hexagonal structure. **(C)** Cubic, pyrochlore-like structure. **(D)** Cubic defect fluorite structure. Note that the cubic form of Dy_2_TiO_5_ may not be fully resolved as of mid-2021. The cubic form of Ho_2_TiO_5_ and Y_2_TiO_5_ consists of a pyrochlore-like structure with a 3x superstructure on (111) planes and a 7x repeat on (622) planes, as described previously (Shepelev and Petrova, 2006, Lau et al., 2007; Lau et al., 2008; Shepelev and Petrova, 2008; Aughterson et al., 2014, Aughterson et al., 2015, Aughterson et al., 2018b).

In comparison to the “227” pyrochlores, following Newman et al. (2018) the “215” type cubic compounds can be reformulated from A_2_BO_5_ to give ^VIII^A_2_^VI^(A_0.667_B_1.333_)^IV^O_6_^IV^O_0.667_, in which A and B are trivalent and tetravalent cations, respectively. This expression is particularly applicable to the A_2_BO_5_ compounds that are related to pyrochlore. As shown in Figure 1, the 215-type compounds adopt orthorhombic, hexagonal, and cubic structures from A = La to Lu and B = Ti. In samples with B = Ti and A = Y or Ho, the A//B ratio of 2/1 leads to a pyrochlore-like structure which exhibits a tripling of the lattice repeat distance on (111) planes, a feature that is not present in samples with other A-site cations or in any of the known compounds with B = Zr. The tripled lattice repeat may be due to a partial ordering of the A and B cations in the structure. We note, however, that electron diffraction patterns taken in the [110] zone axis orientation also show evidence of a 7-fold repeat on the (662) planes, as first described by Lau et al. (2007). This structure evolves to a disordered, defect fluorite with incorporation of progressively smaller cations from Er to Lu. On disordering of the cations and anions, the formula ^VIII^M^IV^X_2-x_ reflects the local coordination environments of the structure in space group *Fm3m* with the anion deficiency determined by the average valence state of the cations, giving x = 0.25 and 1.75 total oxygens in the formula for the 227 type pyrochlore (M/X = 0.5714) and x = 0.333 and 1.667 total oxygens in the formula for the 215 type (M/X = 0.6000). In the disordered defect fluorite structure, the formulae for 227 and 215 type compounds are MX_1.75_ and MX_1.667_, respectively.

## Phase Transitions

In setting the stage for the work to be presented here, it is important to proceed with a fundamental understanding of the pyrochlore to defect fluorite phase transformation and the underlying physical chemistry in relation to changes in stoichiometry as evidenced by intrinsic properties such as the A/B cation ionic radii and numerical ratios and overall cation/anion ratios in these materials. Extrinsic properties—time, temperature, and pressure also have an influence on the phase transformation. For example, during synthesis Gd_2_Zr_2_O_7_ initially crystallizes in the defect fluorite form, possibly as a metastable phase built on a nano-domain model recently proposed by Simeone et al. (2017), but then transforms to an ordered pyrochlore at ∼ 1,250–1,300°C. At higher temperatures up to ∼1,500°C the structure transforms back to the disordered defect fluorite structure (see, e.g., Zhou et al., 2016 and references therein), so in many of these refractory oxide systems it is not uncommon for the thermodynamically stable lower temperature form to be kinetically inhibited during the chemical synthesis and thermal processing stage. Even at high temperature, the reaction rates in these systems may be slow, meaning that assessments of equilibrium should be approached with caution.

**FIGURE 2:**
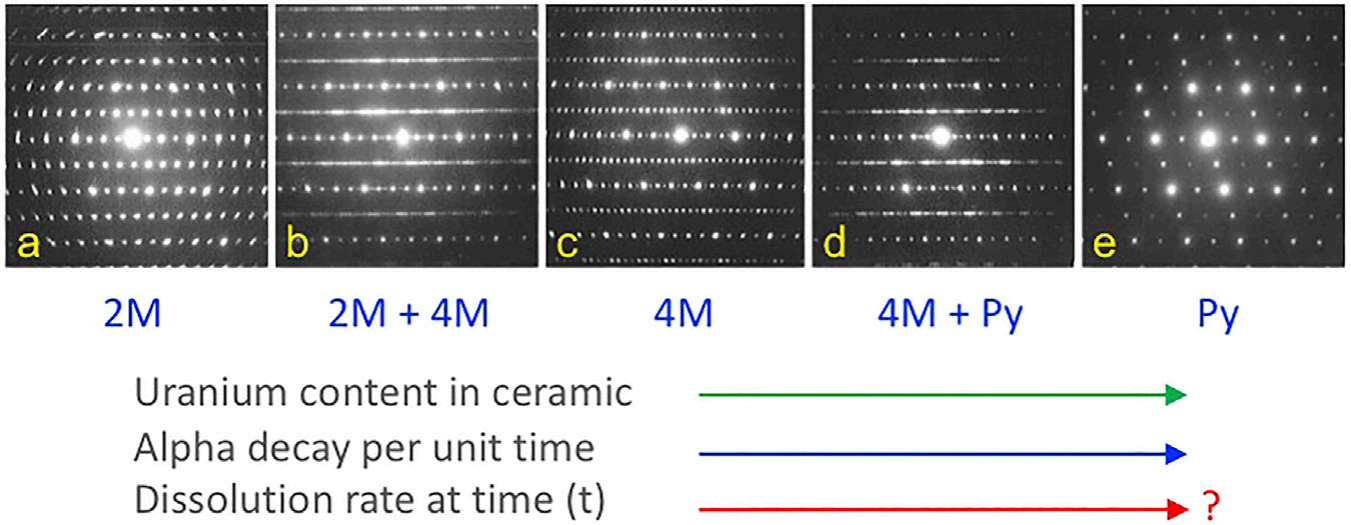
Phase transformations in the series CaZrTi_2_O_7_-CaUTi_2_O_7_ from zirconolite to pyrochlore. From left to right, there are two main zirconolite polytypes (2 and 4M), three single-phase fields **(A, C, E)**, and two intervening two-phase fields **(B, D)** in this system. Similar materials have served as the basis for nuclear waste forms for Pu and other actinides, with reasonably high resistance to aqueous dissolution and release of radioactive components. Adapted from Vance et al. (2002).

As a result of interest in host phases for actinides and fission products in nuclear waste forms, some of the phase transitions from the ABC_2_X_7_ zirconolite compounds to A_2_B_2_O_7_ pyrochlore have been studied in considerable detail. However, these studies have mostly been conducted on ceramic samples as a function of composition with limited temperature variation. Vance et al. (2002) conducted an important study of the incorporation of uranium in zirconolite which built some of the foundations for subsequent nuclear waste form development. This work showed that substitution of U in zirconolite from CaZrTi_2_O_7_ to CaUTi_2_O_7_ resulted in the following phase fields: zirconolite 2M, zirconolite 2M + 4M, zirconolite 4M, zirconolite 4M + pyrochlore, and pyrochlore, as shown in Figure 2. The ability to produce ceramics in this system with fluorite superstructures, high aqueous durability, and considerable compositional flexibility led to the successful development of similar nuclear waste forms for Pu remediation (see Strachan et al., 2005; Icenhower et al., 2006; Strachan et al., 2008). In fact, this system can be tailored for the best balance of waste loading, additives (e.g., neutron absorbers), fission products, an overall aqueous durability (see Lumpkin, 2006, for further discussion and an example of a Pu-based pyrochlore-zirconolite waste form).

**FIGURE 3:**
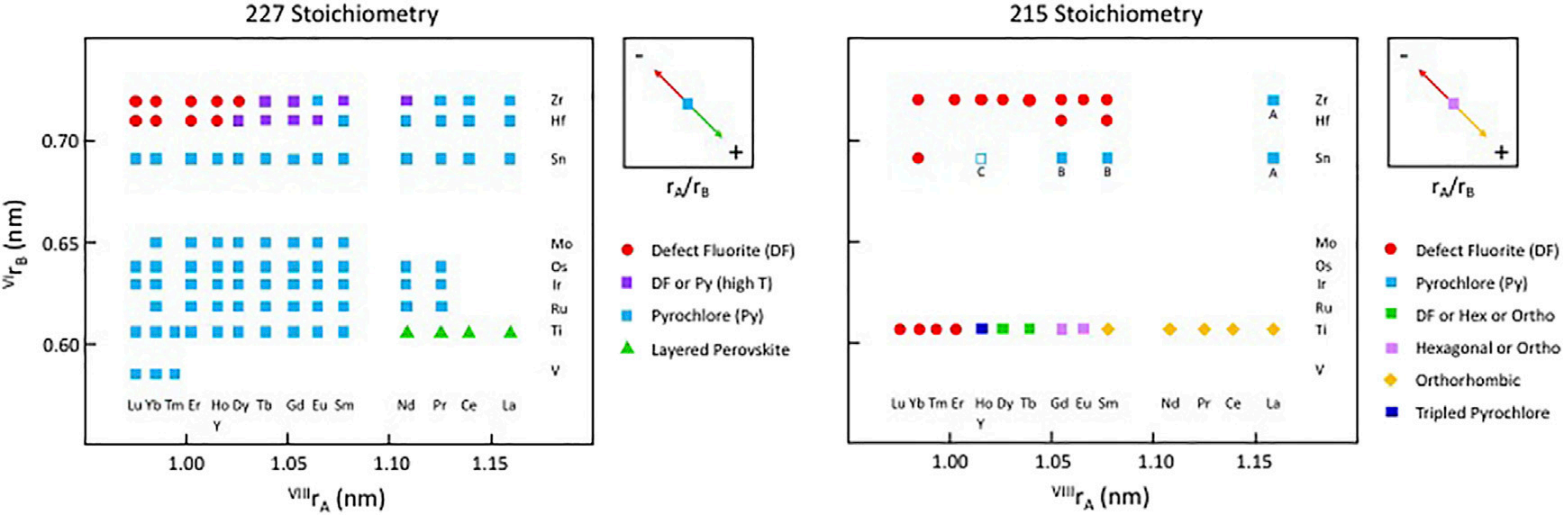
Structure-field map for the A_2_B_2_O_7_ system on the left and a partial structure-field map for some of the known A_2_BO_5_ compounds on the right, both with A = lanthanides and Y and B = selected transition metals at low pressure. This figure does not include the weberite type layered compounds with “227” stoichiometry. Only the series with B = Ti, Sn, Hf, or Zr have been studied fully across the range of A-site constituents and for solid-solutions between both A-site and B-site constituents. The compound Y_2_SnO_5_, is shown with an open square as this may be A-site deficient and multiphase, containing C-type Y_2_O_3_. Other compounds with A = Gd and Sm and B = Sn may contain B-type Gd_2_O_3_ and Sm_2_O_3_, whereas compounds with A = La and B = Sn and Zr may contain the A-type La_2_O_3_ compound as an extra phase (indicated by the letters A, B, or C below the symbols). These latter details remain to be investigated.

**FIGURE 4:**
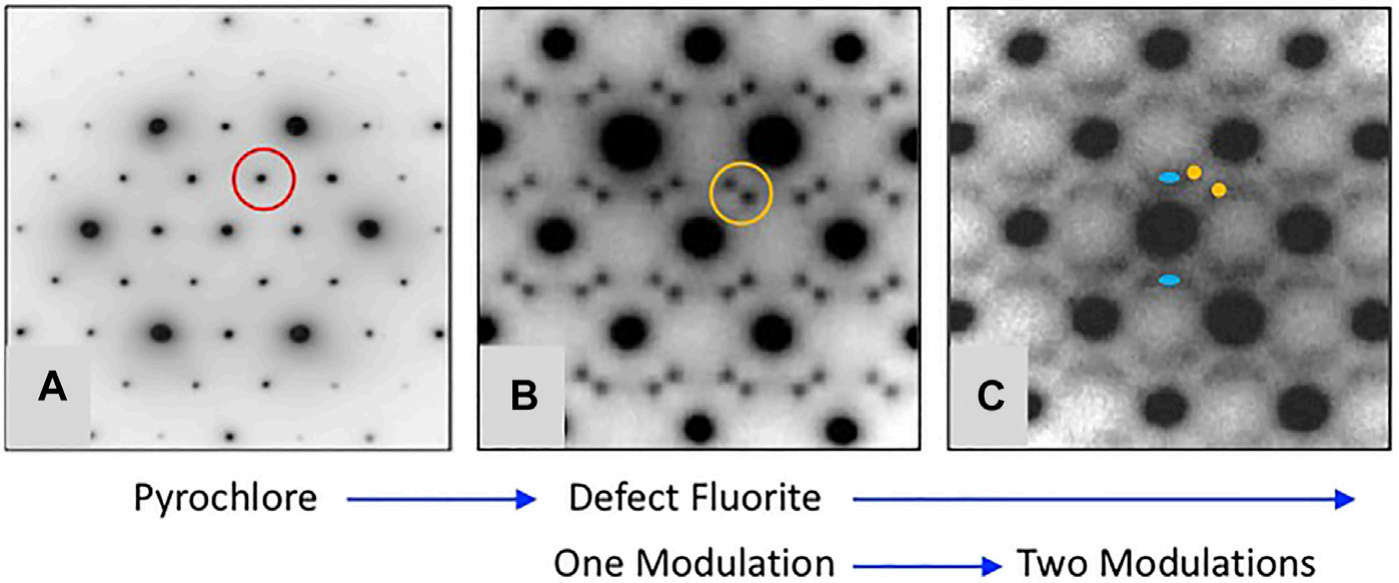
Representative [110] zone axis electron diffraction patterns from A_2_B_2_O_7_ compounds with A = Y and B = Sn and Zr showing the development of modulated structures with increasing substitution of Zr for Sn. Figure is modified from De los Reyes et al. (2013). The single modulation in **(B)** occurs at a moderate level of substitution of Zr for Sn and the characteristic split pair is defined as *G*_*F*_ ± ½ <111>*, e.g., compare the scattering inside the red circle in **(A)** with that inside of the orange circle in **(B)**. Upon further substitution of Zr for Sn the scattering becomes more complex as shown by diffuse streaks and the appearance of a second modulation defined as *G*_*F*_ ± ¼ <220>* and marked by the blue ovals in **(C)**. This sequence of diffraction patterns illustrates a common trend in the pyrochlore to defect-fluorite transformation as a function of r_A_/r_B_ in the various chemical systems.

Figure 3 shows a version of the well-known structure map for the 227 type compounds with A = trivalent Y or La-Lu and B = tetravalent V, Ti, Ru, Ir, Os, Mo, Sn, Hf, and Zr. This structure-field map reveals that the titanates with La-Nd prefer the monoclinic layered perovskite structure (e.g., Harvey et al., 2004 and references therein) whereas the hafnates and zirconates with Lu-Y/Ho prefer the cubic, defect fluorite (DF) structure. This structure is the classical defect fluorite based on space group *Fm*3*m* with cations and anions disordered over the available sites. In between these two-phase fields, we find that most of the compounds in this system adopt the ordered pyrochlore (Py) structure and that a few of the hafnates (A = Dy-Eu) and zirconates (A = Tb-Nd, not including Eu) can be transformed to the disordered defect fluorite structure at high temperature. Within these systems, there have been numerous studies of the phase transformation from pyrochlore to defect fluorite as a function of composition using various combinations of electron diffraction, laboratory-based X-ray diffraction, synchrotron X-ray diffraction and spectroscopy, and neutron scattering (e.g., Withers et al., 1991; Whittle et al., 2009; Reid et al., 2012; Reynolds et al., 2013; Shamblin et al., 2016; Shamblin et al., 2018; Drey et al., 2020). The composition driven phase transformation generally proceeds as a function of the A/B cation ionic radius ratio (e.g., as r_A_/r_B_ decreases) from Py to (Py + DF) to DF. It is well documented in the literature that the DF phase exhibits modulations of the structure as revealed by electron diffraction. An example of this effect is presented in Figure 4 for the Y_2_Zr_x_Sn_2-x_O_7_ series of compounds (De los Reyes et al., 2013). With increasing Zr content in this series, the structure transforms from pyrochlore to defect fluorite via a narrow two-phase field, initially showing one modulation (*G*_*F*_ ± ½ <111>*) of the DF phase which appears as a pair of sharp satellite reflections. With increasing substitution of Zr for Sn, this modulation becomes diffuse and a second modulation appears (*G*_*F*_ ± ¼ <220>*). This appears to be a characteristic feature of Py-DF solid solutions approaching the C-type rare earth oxide stability field. Using the same series of compounds, Zhang et al. (2013) used a combination of synchrotron X-ray diffraction, X-ray absorption spectroscopy, and *ab initio* computer simulations to show that a distinct phase transition from ordered pyrochlore to disordered defect-fluorite occurs at *x* ∼ 1.0–1.2. However, X-ray absorption near-edge structure (XANES) data for the Zr L_3_ and Y L_2_-edges demonstrated a gradual evolution of the structure over the entire compositional range. Therefore, it is apparent that the local disorder begins long before the pyrochlore to defect-fluorite phase boundary is reached and continues to develop throughout the defect-fluorite region.

For comparison, Figure 3 also shows a similar structure map for the 215 type compounds based on the same set of A and B-site cations. The knowledge base for compounds with this stoichiometry has been developed only for the compositions with B = Ti and, to a lesser extent B = Zr, with a few additional data points for B = Sn. Excluding the effects of cooling rate and pressure, the information presented here is reasonably complete for samples with B = Ti. The available data show that the A_2_TiO_5_ compounds are orthorhombic for A = La-Sm; orthorhombic or hexagonal for A = Eu and Gd; orthorhombic, hexagonal, or cubic (defect fluorite) for Tb and Dy; tripled pyrochlore for Ho and Y; and defect fluorite structures for Er-Lu. For these compounds, the phase diagram of Petrova et al. (1982) is the current benchmark for the high temperature behaviour. The topology of this phase diagram is the basis for much of the recent work in providing a detailed description of phase fields as a function of composition and temperature. The phase diagram shows that the melting points of the various compounds increase from about 1,600 to 2,200°C with decreasing ionic radius from La through to Yb. The topology of the phase diagram indicates that the solid-state phase transitions with increasing temperature are likely to proceed from orthorhombic to hexagonal to cubic within a narrow range of ionic radii of the trivalent lanthanide cations, e.g., approximately in the region from Sm to Er. To date, only a few other 215-type compounds have been synthesized with other B-site cations, including samples with B = Sn, Hf, and mainly Zr. The compounds shown in Figure 3 with B = Zr and A = Sm-Yb, together with Sm_2_HfO_5_ and Gd_2_HfO_5_, are single-phase defect fluorites, whereas a production run with A = La was not stoichiometric, having led to the formation of A-type La_2_O_3_ as a second phase. Other samples produced with B = Sn also contained a second phase, either A-type or B-type sesquioxide depending on the particular lanthanide cation on the A-site. Microanalysis of the run product for Y_2_SnO_5_ indicated that it had both A-site vacancies and C-type Y_2_O_3_ as a second phase (see Newman et al., 2018).

**FIGURE 5:**
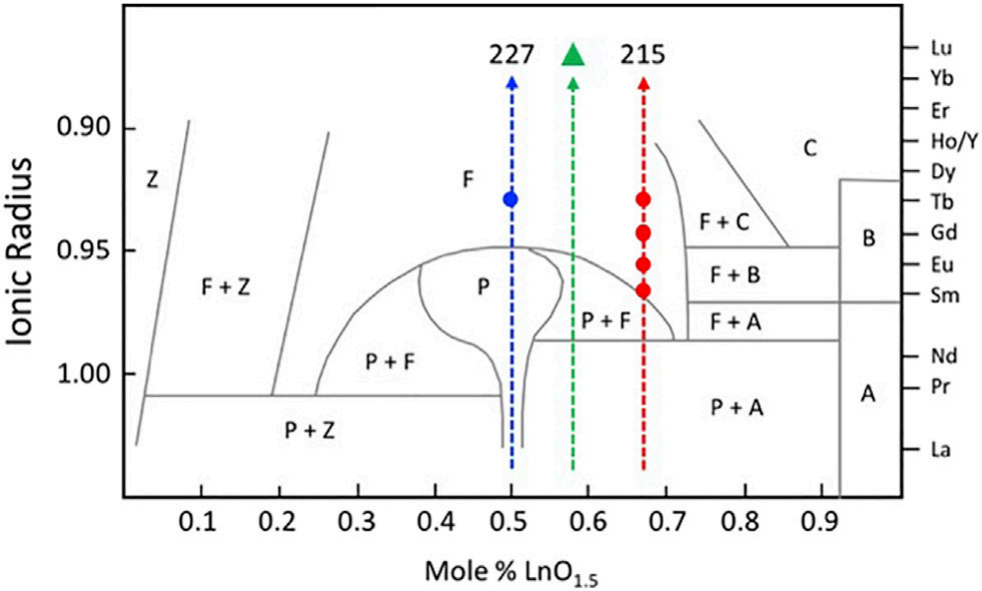
Phase fields found in the ZrO_2_—Ln_2_O_3_ system. This figure is adapted from Tabira et al. (2001). Z = ZrO_2_, F = defect fluorite, P = pyrochlore, A = hexagonal A-type Ln_2_O_3_, B = monoclinic B-type Ln_2_O_3_, and C = C-type Ln_2_O_3_ (cubic bixbyite structure). The vertical dashed lines mark the stoichiometries of the 227, delta phase (green triangle), and 215 compounds in this system. The solid blue circle shows the location of the Tb_2_Zr_2_O_7_ defect fluorite phase. The solid red circles show the locations of the Sm_2_ZrO_5_, Eu_2_ZrO_5_, Gd_2_ZrO_5_, and Tb_2_ZrO_5_ defect fluorite phases (see Figures 6, 7 for a discussion of the diffuse scattering features in these compounds resulting from modulations of the parent defect fluorite structure).

**FIGURE 6:**
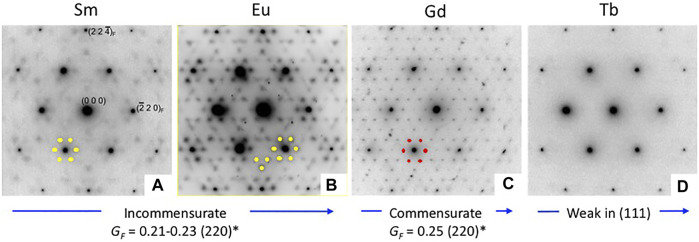
Electron diffraction patterns taken for the [111] zone axis for Sm_2_ZrO_5_ **(A)**, Eu_2_ZrO_5_ **(B)**, Gd_2_ZrO_5_ **(C)**, and Tb_2_ZrO_5_ **(D)**, showing the gradual changes in the additional scattering features in these compounds with decreasing A-site cation radius from left to right. Modulations of the underlying defect fluorite structure are indicated by the weak beams surrounding the strong ones. Note the gradual change in the modulation of (220)* and symmetry related beams from Sm to Tb. Does the (apparently) commensurate (4x) structure in Gd_2_ZrO_5_ indicate ordering in the (111) plane? Note also that the extra diffraction spots are nearly absent in Tb_2_ZrO_5_.

**FIGURE 7:**
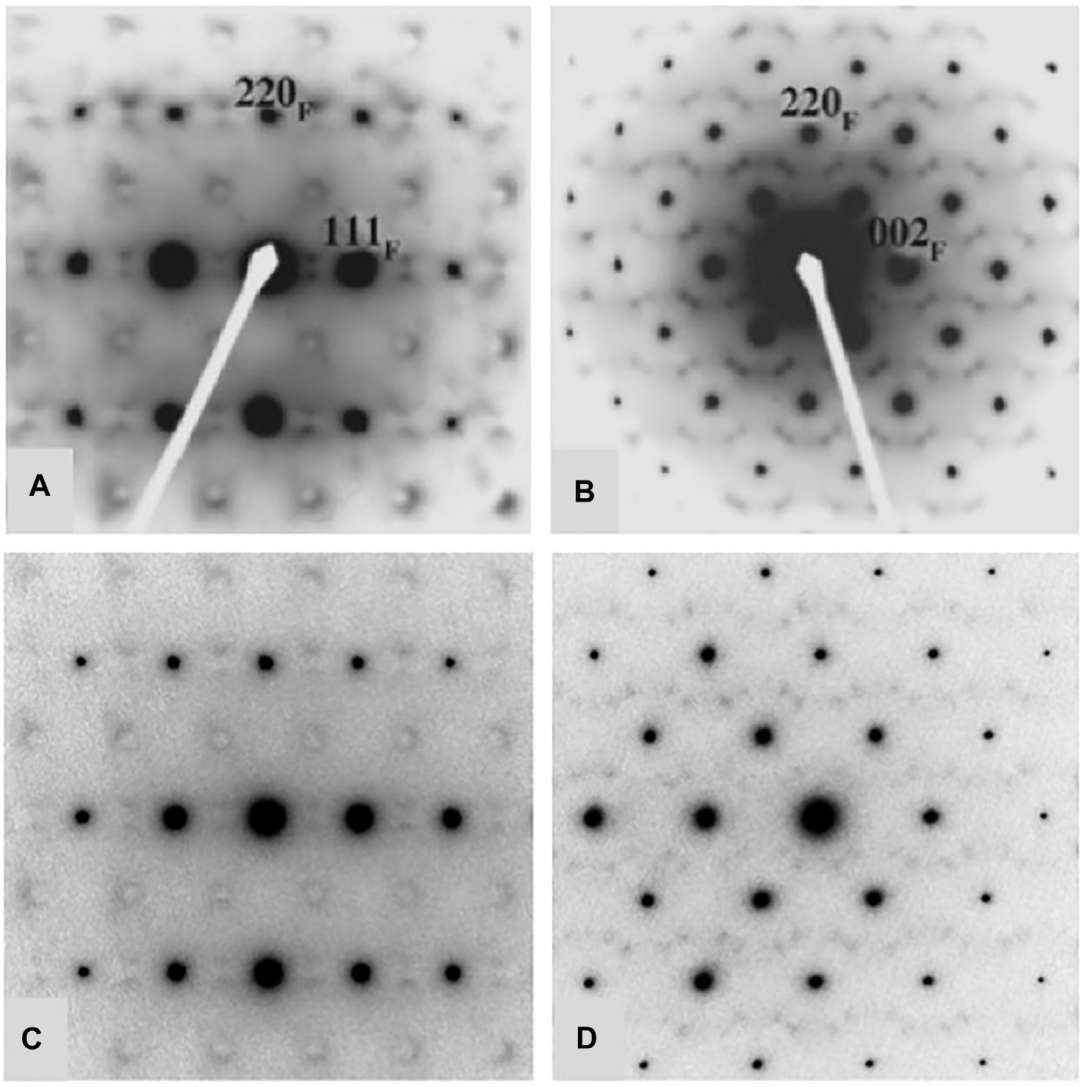
Comparison of diffuse scattering features in Tb_2_Zr_2_O_7_ **(A,B)** and Tb_2_ZrO_5_ **(C,D)**. Both compounds are defect fluorite structure types showing characteristic modulations the [112] zone axis in **(A,C)** and the [011] zone axis in **(B,D)**. However, in spite of the major difference in composition (A/B cation ratio and number of anion vacancies), the diffuse features and their measured wave vectors are identical within error. Figures **(A,B)** are after Tabira et al. (2001).

A considerable amount of additional information related to the “stability” fields of defect fluorite, pyrochlore, and their component oxides is available in the phase diagram (or structure-field map) presented by Tabira et al. (2001), reproduced here in slightly modified form in Figure 5. This figure was developed specifically for the Ln_2_O_3_-ZrO_2_ system of compounds and shows the various single phase and two-phase fields as a function of the LnO_1.5_ content (mole %) on the *x*-axis versus the ionic radii of the Ln cations on the *y*-axis. This particular diagram provides an excellent basis for understanding the nature of the defect fluorite phase and the types of modulations that occur as a function of composition in the single-phase region and in two-phase regions next to the phase field of zirconia, on either side of the pyrochlore field, and next to the phase fields of the A, B, and C-type rare earth oxides. This work helped to underpin the concept of a Py to DF transformation driven by strain in the close-packed {111} cation layers of the DF structure by cation and oxygen vacancies. In Figure 5, we also plot the locations of four 215-type compounds with A = Sm, Eu, Gd, and Tb (red dots). The structural complexity of these compounds is illustrated in Figure 6, showing the gradual evolution of the modulations in their [111] zone axis electron diffraction patterns. With decreasing ionic radius of the A-site cation, these compositions transition away from the pyrochlore + fluorite two-phase field and presumably toward the fluorite + C-type Ln_2_O_3_ field. On proceeding from A = Sm through to Tb, the modulations become stronger and transition from incommensurate to (apparently) commensurate for A = Gd, but they are very weak and diffuse for A = Tb. Finally, we note also that the structural modulations for Tb_2_Zr_2_O_7_ and Tb_2_ZrO_5_ in the [110] zone axis are virtually identical, indicating that the stoichiometry change from 227 to 215 type has not changed the modulation wave vector significantly (Figure 7). This is another interesting problem for the detailed interpretation of strain-driven transformations in this system as a function of the Tb/Zr ratio.

## Radiation Tolerance

The radiation tolerance of the large group of fluorite and related compounds has been studied extensively for the MX_2_ fluorite compounds and the A_2_B_2_O_7_ pyrochlore and defect fluorite compounds. Furthermore, there is a growing body of evidence in relation to the radiation tolerance and other properties of the A_2_BO_5_ compounds. This will be discussed briefly here for several different experimental methods. Atomistic simulations investigate the performance of the material on picosecond time scales through an analysis of the energetics of defect formation and migration (e.g., Minervini et al., 2000; Jiang et al., 2009). In the latter context, one may generally consider that radiation tolerance is promoted by high defect formation energies and low energy barriers to atomic migration in the host structure. In this situation, it is more difficult to move an atom out of its normal crystallographic site, but if enough energy is imparted to the atom to displace it, then it may easily return to either the original site or a similar one nearby. On the other hand, if energy barriers to migration are high, it is more likely that radiation-induced displacements and damage will be retained under a given set of conditions (time, temperature, etc.). These principles have been demonstrated by a combination of theoretical and experimental work over the course of 30 years or more. A major outcome of the modelling work on compounds with A_2_B_2_O_7_ stoichiometry is that pyrochlore and defect fluorite compounds exhibit lower cation antisite and anion Frenkel defect formation energies as a function of the A/B cation radius ratio r_A_/r_B_. As the value of r_A_/r_B_ decreases, the structures may transform from pyrochlore to a defect fluorite structure as a function of temperature or possibly as a result of radiation damage.

Many ion irradiation experiments have now been completed where the experimental time is on the scale of minutes or hours (e.g., Begg et al., 2001; Aughterson et al., 2016, Aughterson et al., 2018a; Aughterson et al., 2018b; Park et al., 2018; Whittle et al., 2011). These high dose rate experiments have provided much of the fundamental physics knowledge on radiation damage in fluorite and derivative materials as a function of ion mass, energy, and temperature. Major conclusions to be drawn from these studies indicate that the A_2_B_2_O_7_ pyrochlore and defect fluorite compounds are increasingly radiation tolerant (e.g., high critical amorphization dose and low critical amorphization temperature) as a function of the A/B cation radius ratio (r_A_/r_B_). Because the A-sites are devoted to a given set of nuclear waste elements, the B-sites are generally available to host larger cations like Hf and/or Zr, which increase the value of r_A_/r_B_ over that of similar pyrochlores with B = Ti (and also provide the potential to improve both the chemical durability and the resistance to amorphization). As a result, a particular group of defect fluorite compounds, e.g., those with B = Zr and A = smaller lanthanides, tend to remain crystalline under irradiation. In the real world, of course, these materials must include actinides and possibly some fission products and impurity elements and maintain a similar level of radiation resistance and chemical durability for optimum performance under wasteform storage conditions. Although the A_2_BO_7_ compounds with B = Ti are not radiation tolerant, the orthorhombic structures do exhibit critical amorphization temperatures that are lower than those the corresponding A_2_B_2_O_7_ pyrochlore and defect-fluorite compounds. Additionally, the A_2_BO_7_ type compounds have the higher A/B cation radius ratio of 2:1, in theory allowing higher waste loadings. Finally, the defect-fluorite A_2_BO_7_ compounds with B = Zr may have the potential to provide both higher waste loadings, neutron absorbers for criticality control, and “built-in” radiation tolerance.

Unfortunately, only a few candidate materials have been doped with short-lived actinides such as ^244^Cm (t_1/2_ = 18.1 y) or ^238^Pu (t_1/2_ = 87.7 years) where the experiments generally run for less than 5 years and are highly relevant to the development of nuclear waste forms (e.g., Weber 1998; Strachan et al., 2005, 2008). This work has demonstrated that waste forms based on pyrochlore and/or zirconolite become amorphous with increasing dose with the potential for volume expansion (on the order of 5%) and cracking, thereby increasing the surface area of the waste form. Finally, there have been a few investigations of natural analogues containing long-lived ^235^U (t_1/2_ = 7.0 × 10^8^ y), ^238^U (t_1/2_ = 4.5 × 10^9^ y), and ^232^Th (t_1/2_ = 1.4 × 10^10^ y), including pyrochlore, zirconolite, and other radioactive minerals. These studies demonstrate that pyrochlore and zirconolite become amorphous with increasing alpha-decay dose. However, it appears that zirconolite and, to a lesser extent, pyrochlore, are capable of actinide retention in all but the most severe conditions imposed by nature. We note here that there are no Zr-rich natural pyrochlores, as the pressure, temperature, and bulk rock compositions generally dictate that ZrO_2_ (baddeleyite) is a stable phase in the mineral assemblages with low *a* (SiO_2_), whereas zircon is stable in assemblages with high *a* (SiO_2_).

Finally, we note here that the radiation damage effects and physical/mechanical properties of natural pyrochlores have recently been studied in some detail in relation to percolation theory, although the compositions of these samples differ significantly from the target compositions of pyrochlore-based nuclear waste forms (e.g., the natural samples may have significant amounts of Na on the A-site, Nb and Ta on the B-site, together with F and OH on the Y-site). Nevertheless, the results are significant in providing a fundamental basis for understanding the physical properties of pyrochlore based materials under irradiation. Firstly, Zeitlow et al. (2017) presented a new study of the thermal annealing behaviour and mechanical properties of natural pyrochlore samples with amorphous fractions ranging from about 8 to 100%, showing how the recovery steps of the three most damaged samples systematically increase in temperature as a function of the initial damage fraction. This work included detailed analyses of the Raman and infrared spectra of these samples. Furthermore, Reissner et al. (2020) provided additional of evolution of the hardness and elastic moduli of three of the same samples as a function of the annealing temperature and time (16 h in air, per temperature step). A further assessment of the mechanical behaviour of the pyrochlores was conducted by Beirau and Huber (2021) by using finite element-voxel models the calculate the elastic moduli and hardness values as a function of alpha-decay dose and annealing temperature. It is noteworthy that the implied, model dependent percolation points at ∼16% (p1) and 84% (p2) of the amorphous fraction are reasonably consistent with previous experimental work on radiation damaged pyrochlores (Lumpkin and Ewing, 1988).

## Opportunity Space and Questions

Based on this brief look at defect fluorite and related compounds and systems, it is apparent that there remain considerable opportunities for ongoing research and development within the very large composition space indicated by Table 1. Future research opportunities include the fundamental physical chemistry of phase transitions from the various ordered structure types listed here to their disordered defect fluorite structures or from one ordered structure to another. Knowledge generated from an understanding of the basic crystal chemistry and physical chemistry across these systems opens the door to novel materials research and development, in this case, there are many potential applications in areas such as the high-technology sector and energy/nuclear materials that might benefit from further fundamental research. For these and other applications, future work might be devoted to the development of specialist materials taking advantage of the properties of structure types such as defect fluorite, pyrochlore, weberite, delta phase, and 215-type compounds in the design, production, and testing of new materials. In particular, one area of interest may be the design and testing of composite materials using various combinations of these structure types, provided that phase compatibility can be achieved via both chemical and dimensional compatibility.

In particular, Figure 3 indicates that there are considerable opportunities for research to be conducted within the composition space for the A_2_BO_5_ compounds. Currently, most investigations have focused on the compounds with B = Ti, with more recent efforts devoted to the materials with B = Zr and, to a lesser extent with B = Sn and Hf. However, there have been few, if any, investigations into the systems with B = Mo, Os, Ir, Ru, and V for this particular stoichiometry. Even in the structures with B = Ti, there remain some uncertainties in the structures, e.g., as suggested by electron diffraction patterns that might lead to questions about the actual symmetry or in the details related to incommensurate, modulation effects and their origins. The fundamental physical chemistry of new materials with B = V, Ru, Rh, Ir, Os, and Mo (and possibly other elements, e.g., Sb, Nb, Ta, and Pb and Bi in their different valence states) together with new investigations of samples with B = Sn, Hf, and Zr. How do the orthorhombic, hexagonal, cubic, and other structures (e.g., Shepelev and Petrova, 2006, Shepelev and Petrova, 2008) evolve in these systems as a function of composition and what are their properties? Furthermore, there is considerable scope for new studies to be conducted on the high temperature behavior in the system with B = Ti plus variable amounts of other small cations in a substitutional role.

Another opportunity space will involve the future study of structural evolution between the A_2_B_2_O_7_ compounds and their A_2_BO_5_ counterparts. Similar comments apply more broadly to some of the other structure types where we may find a wealth of new information (and new functional materials?) in the study of phase transitions between the various delta phase, 215-type, 227-type, and 317-type compounds listed in Table 1. An interesting aspect of this problem is the wide variation in composition and stoichiometry (M/X = 0.5–0.6 and X vacancies = 0.0 to 0.5 per M cation) found in the different groups of compounds. Furthermore, the observation in this paper that Tb_2_Zr_2_O_7_ and Tb_2_ZrO_5_ exhibit identical modulations of the underlying defect fluorite structure suggests that such modulations are not a precise function of the Tb/Zr ratio, M/X ratio, or the number of oxygen vacancies (0.250 and 0.333), respectively, relative to the disordered MX_2-x_ fluorite basis. In general terms, the origin and significance of these unusual crystallographic features presents another interesting topic for future experimental and theoretical work. For example, just what is the fraction of atoms involved in both displacive and compositional components of the modulations and how is this related to the populations of defects discussed below?

Following from the above discussion of modulations, it is of utmost importance to understand the defect chemistry of the fluorite structure and its more complex derivative compounds in greater detail in order to complement studies of physical-chemical properties and radiation damage effects. In ordered pyrochlore, for example, these defects are generally understood to be a combination of cation anti-site (CA) defects, cation Frenkel (CF) defects, and anion Frenkel (AF) defects. The transition from pyrochlore to defect fluorite is related to the energetics of the CA and AF defects and has been mapped out in some detail using atomistic simulations (e.g., Minervini et al., 2000; Jiang et al., 2009; Uberuaga et al., 2015; Archer et al., 2021) as a guide to predict order-disorder trends in these compounds as a function of the ionic radii of the A and B cations. In general, the defect formation energies of CA and AF defects decrease as the ionic radius ratio r_A_/r_B_ decreases and this in turn corresponds to the transformation from pyrochlore to defect fluorite structures in numerous solid solutions. Together with fundamental crystal chemistry parameters, the simulations noted here were also used to develop a predictive model of radiation tolerance in pyrochlore and defect fluorite compounds (Lumpkin et al., 2007). In general terms, it would be very interesting to compare the radiation damage data for A_2_B_2_O_7_ and the cubic A_2_BO_5_ compounds with the same A and B cations to explore the effect of anions vacancies on damage recovery, if any. Finally, we suggest that considerable improvements might be made in the study of defects in these compounds through a combined modelling and experimental approach directed toward an advanced interpretation of, for example, neutron pair distribution functions with and without specific defects. Furthermore, can the effects of incommensurate structures or layer stacking disorder be incorporated into this problem in a quantitative way? This approach would necessarily involve the calculation of the relevant scattering functions for different structure types with and without an array of defects such as interstitials, vacancies, cation anti-site arrangements, Frenkel defects, and stacking disorder within the host crystal.
